# Engaging Communities and Empowering Research: Lessons from a Network of Community Engagement Cores

**DOI:** 10.3390/ijerph22111661

**Published:** 2025-11-01

**Authors:** Daniel F. Sarpong, Corrine Yibing Liu, Tandeca King Gordon, Angela Sy, Bibiana Mancera, Mustapha Alhassan, for the RCMI Community Engagement Consortium

**Affiliations:** 1 Office of Health Equity Research, Yale School of Medicine, Yale University, New Haven, CT 06519, USA; corrine.liu@yale.edu; 2 RCMI Coordinating Center, Morehouse School of Medicine, Atlanta, GA 30310, USA; tgordon@msm.edu; 3 Department of Tropical Medicine, Medical Microbiology, and Pharmacology, John A Burns School of Medicine, University of Hawaii at Manoa, Honolulu, HI 96813, USA; sya@hawaii.edu; 4 Border Biomedical Research Center, University of Texas at El Paso, El Paso, TX 79968, USA; barias@utep.edu; 5 Whitney M. Young, Jr. School of Social Work, Clark Atlanta University, Atlanta, GA 30310, USA; malhassan@cau.edu

**Keywords:** community engagement, health disparities, community-based participatory research (CBPR), minority health, underrepresented populations, academic–community partnerships, health equity, research translation, capacity building

## Abstract

As of the end of 2024, the NIH-funded Research Centers in Minority Institutions (RCMI) Program supported 21 specialized centers at minority-serving institutions. Its goal is to strengthen biomedical research infrastructure and enhance the capacity of investigators focused on health disparities. Since 2017, the centers have operated under a unified framework that includes four core components, one of which is Community Engagement (CEC). In 2021, the RCMI Coordinating Center established the CEC consortium to unify expertise across minority-serving institutions, which include historically Black colleges and Hispanic-serving institutions. This consortium promotes cross-institutional collaboration and innovation in community-engaged research to advance health equity. This paper examines how RCMI CECs strategically enhance research relevance, advance public health outcomes, and address social determinants of health (SDOH) through inclusive, bidirectional partnerships that position communities as co-leaders in the research process. Drawing on data from NIH RePORTER, the 2024 Community Engagement Consortium Signature Programs Monograph, and RCMI Common Data Elements, we analyze the collective contributions of the Community Engagement Core (CECs) across 21 RCMI centers. Findings underscore the role of tailored strategies, cultural competence, and academic-community partnerships in mitigating health disparities and promoting equity in underserved communities.

## 1. Introduction

### 1.1. RCMI U54 Centers

The Research Centers in Minority Institutions (RCMI) program was established in 1985 under the National Institutes of Health (NIH) National Center for Research Resources and was transferred in 2017 to the National Institute on Minority Health and Health Disparities (NIMHD). It is a coordinated NIH U54 program designed to expand research infrastructure, support investigator development, and strengthen community-engaged research at minority-serving institutions. It was in 2017 that the NIH RCMI Request for Applications (RFAs), starting with RFA: PAR-11-132, established the Community Engagement Core, which became a required core. The RCMI program aims to advance minority health and health disparities research, develop a diverse biomedical research workforce, and promote sustainable community partnerships at institutions committed to educating underrepresented students and serving medically underserved communities [1]. Through its structured core model, RCMI centers implement institution-level strategies aligned with national priorities for health equity. By prioritizing diseases disproportionately affecting minority populations, RCMIs strengthen the national capacity for biomedical, behavioral, and clinical research while addressing health inequities.

In 2024, the RCMI program supported 21 research centers. Although NIH/NIMHD sets broad priorities, the specific activities of each CEC—including policy advocacy—are shaped in collaboration with community partners through advisory boards, needs assessments, and ongoing dialogue. This participatory structure enables each center to respond to local community priorities while contributing to the NIMHD’s national mission of advancing health equity through research, training, and community engagement. Although the collective goals of the RCMI program have undergone some changes as of 2024, with 21 funded U54 Centers, the collective goals of the RCMI Program were as follows:

1. Enhance Institutional Research Capacity: Strengthen the infrastructure to conduct cutting-edge basic biomedical, behavioral, and clinical research.
2. Increase Investigator Competitiveness: Support investigators at all levels to obtain competitive extramural funding, particularly for research on diseases and conditions disproportionately impacting minority and health disparity populations.
3. Foster Career Development: Create environments conducive to the career advancement of postdoctoral fellows, junior faculty, and early-stage investigators.
4. Advance Minority Health Research: Enhance tools, methodologies, and dissemination of research findings to improve minority health and reduce health inequities.
5. Build Community Partnerships: Establish sustainable relationships with community-based organizations to address health-related concerns, promote research participation, and disseminate findings effectively.
6. Support Biomedical Workforce: Train and mentor the next generation of researchers from all backgrounds to advance health equity.
7. Promote Collaborative Research: Facilitate consortia and partnerships across institutions to drive innovation and impactful research in health disparities.

### 1.2. RCMI Community Engagement Cores Goals and Functions

With the transition of the RCMI program’s administrative oversight from the National Center for Research Resources (NCRR) to the NIMHD in 2017, Community Engagement Cores (CECs) became a required structural component to ensure meaningful collaboration with communities served by RCMI [2]. This shift formally acknowledges that addressing health disparities necessitates sustained, intentional engagement with the communities most affected [3]. CECs ensure that research reflects local priorities and lived experiences, aligning with the National Academy of Medicine’s recommendation that community stakeholders be involved in all phases of research [4]. Community engagement not only enhances the cultural relevance and acceptability of research but also strengthens trust and accountability between institutions and the populations they serve [5]. In operationalizing, CECs adopt principles of Community-Based Participatory Research (CBPR), fostering bidirectional relationships between researchers and community partners through shared planning, implementation, and dissemination of research initiatives [5]. Although each CEC may define its own site-specific priorities, the goals across all 21 RCMI converge around seven common functions:

1. Establish and sustain long-term collaborations with community organizations and partners to address health disparities, promote research participation, and ensure community-driven input into health-related research (Community-Based Partnerships).
2. Enhance the cultural competency of researchers and build community capacity in research through training, mentoring, and resource sharing to ensure effective community engagement in health disparities research and intervention development (Capacity Building & Training).
3. Develop strategies to improve inclusivity in recruitment and retention in biomedical and health disparities research, ensuring sustained community participation (Recruitment and Retention).
4. Translate research findings into actionable community-level practices and policies by disseminating results through accessible formats like health education materials, workshops, and community events (Research Translation & Dissemination).
5. Implement ongoing evaluation of community engagement efforts, using surveys, interviews, and other methods to assess the impact of engagement strategies and ensure continuous improvement (Evaluation & Feedback).
6. Foster trust-based relationships between academic researchers and communities, addressing past concerns about research and creating a foundation for sustained and productive partnerships (Building Trust & Reducing Distrust in Research).
7. Leverage innovative and necessary data on social determinants of health (SDOH) and genomic indicators to create databases that enhance personalized, precision healthcare and improve treatment outcomes for minority populations (Data Integration & Precision Treatment).

### 1.3. Importance of Community Engagement in Research

Effective community engagement is critical for research on health inequities, with RCMI Community Engagement Cores (CECs) promoting both scientific rigor and social responsiveness. Engagement occurs along a continuum from outreach to shared leadership, using varied strategies to build equitable partnerships. Israel et al. showed the value of such partnerships in the Neighborhoods Working in Partnership project, which empowered communities and trained partners in advocacy [6]. Likewise, Viswanathan et al.’s review of 185 studies underscored the need to balance strong community–institution relationships with rigorous methods, offering guidance relevant to CECs [7]. Key elements of successful partnerships include building trust [8], centering equity [9], leveraging cultural wealth [10], and cultural sensitivity [11]. Drawing on these principles, CECs employ diverse approaches that strengthen community capacity, ensure research relevance, and advance health equity.

### 1.4. The Unique Role of CECs in Addressing Health Disparities and Building Trust

In 2021, the RCMI Coordinating Center launched the CEC Consortium to unify efforts across the 21 institutions and accelerate progress in community-engaged health disparities research. The Consortium was created to foster cross-site collaboration, promote shared learning, and scale effective community engagement practices. Its central aim is to strengthen the collective capacity of RCMI programs to build and maintain trusted, bidirectional partnerships with the communities they serve, essential mechanisms for reducing health disparities at both local and national levels. Through regular convenings, shared resources, and strategic alignment, the Consortium has identified best practices in community engagement (CE), encouraged co-learning, and advocated for collaboration across institutions. Key initiatives include the development of an operational definition for community-engaged health disparities research (HDR) and a compendium of signature strategies and initiatives implemented by RCMI CECs to improve recruitment, retention, and community participation [12].

### 1.5. Objectives

This descriptive paper catalogs and compares community engagement strategies and outcomes across 21 RCMI Community Engagement Cores, highlighting both similarities and differences through the lens of the CEC Consortium. The objectives are threefold: (1) To describe the core strategies employed across the RCMI CECs to foster community engagement in research, (2) to explore variations in CE approaches across the RCMI centers, and (3) to assess the collective impact of CEC initiatives in communities or populations that they engage with and serve. Findings from this analysis may inform other institutions seeking to strengthen trust, engagement, and capacity in community-based research aimed at addressing health inequities.

## 2. Materials and Methods

### Data Sources

The primary sources of data for this paper were abstracts of each of 21 RCMI Community Engagement Cores (CECs) accessed in the NIH Reporter [13], the Community Engagement Consortium Signature Programs monograph published in 2024 [12], and common data elements (CDEs) captured from two of 13 common metrics (CMs) from the RCMI Program Evaluation Framework. The 2024 Monograph of CEC Signature Programs included one-page stories for each of the two strategies or initiatives from each of the 21 CECs [12]. CEC leaders at each RCMI were asked to identify and write about two strategies or initiatives that they deemed outstanding, successful, and/or unique and might set them apart or serve as a “signature” for that CEC. Community partners were not directly involved in the conduct of this national-level descriptive analysis; instead, the analysis relied on documentation and reports provided by each CEC regarding their local engagement activities. This approach was selected due to the scope and nature of the data, which were aggregated across multiple institutions and sites.

The development of the RCMI CDEs was facilitated by the RCMI Coordinating Center, working in collaboration with evaluators or administrative core staff at each RCMI. Of the CDEs collected across RCMIs, two of the 13 CMs addressed the outcomes of the CECs. Data for CM7—“Number and Type of Academic–Community Partnerships” included the following data elements: the number of formal and informal partnerships (with or without memoranda of understanding or subawards, respectively), new partnerships, and duration of existing partnerships (2–5 years, 6–10 years, and over 10 years). CM8, “Community-Engaged Research—Partnership,” tracks the number of formal and informal community partners (both with and without memoranda of understanding), their distinct roles, the volume of community-engaged research (CEnR), CEnR led by early-stage investigator (ESI) principal investigators (PIs), CEnRs associated with RCMI pilot projects, and CEnR projects externally funded.

## 3. Analysis Approach

### Data Analysis

We conducted a mixed-methods (qualitative and quantitative) analysis using a convergent parallel design to describe the activities, results, and best practices of the 21 CECs both descriptively and numerically. Qualitative and quantitative data were collected simultaneously but analyzed separately, then merged and integrated as the results [14]. 

For qualitative analysis, ChatGPT4 has been demonstrated to be a valuable analytical tool, enhancing the efficiency of thematic analysis and providing additional insights into the qualitative data [15]. A qualitative content analysis was performed to identify themes and subthemes within the RCMI Community Engagement Core (CEC) abstracts available on NIH RePORTER and described in the 2024 CEC Signature Program Monograph [12,13]. The analysis proceeded in two parallel processes: manual coding and AI-assisted coding. For the manual analysis, a team of two coders independently reviewed all abstracts using an inductive approach. Each coder performed open coding to capture key concepts, followed by axial coding to group related concepts into preliminary categories. The team held meetings to reconcile any differences and reach a consensus on the final coding, which included clearly defined themes and subthemes. In the AI-assisted analysis, artificial intelligence tools—including ChatGPT and Notebook LM—were used to generate preliminary summaries, suggest potential categories, and identify semantic relationships across the abstracts. These outputs were considered decision-support tools rather than final results. The human coding team compared the AI-suggested themes with the manually developed codebook, using areas of convergence to validate the findings and areas of divergence to stimulate further discussion. This dual approach allowed triangulation of results, leveraging the efficiency of AI-assisted synthesis while maintaining the interpretive depth and contextual sensitivity of human qualitative analysis.

The 21 CEC abstracts were extracted from the NIH Reporter and combined in a single EXCEL file. The 21 abstracts were manually reviewed, with notes taken on the main themes at the institutional level. In an iterative manner, prompts derived from the manual review of the abstracts were created in ChatGPT and Notebook LM to derive institutional-level themes and then synthesize them into program-level themes. Both AI tools, coupled with manual coding, were used to ensure credible results.

An example of a truncated ChatGPT Prompt is “Use the information provided in the abstract of each institution’s CEC in the attached document to perform the following. Identify the population (community) served, stakeholder involvement, and cultural relevance of their activities or functions. Describe activities and programs such as community advisory boards, workshops and training, and collaborative planning to strengthen community partnerships. Identify strategies like transparency, shared leadership, and cultural competence to build trust with the target community or population.” We generated word clouds to visually represent themes related to community engagement and CBPR principles. This content analysis also identified community engagement strategies that are both common and unique across the individual CECs within the consortium, mapping each strategy to the institutions that employ it.

To contextualize the CECs’ engagement activities and their intensity, we used the CDC’s Spectrum of Community Engagement framework, with the range: Outreach → Consult → Involve → Collaborate → Shared Leadership. Summary statistics (frequency distributions) were calculated using the CDEs for the period 2022–2023 related to the two common metrics: CM7—“Number and Type of Academic–Community Partnerships” and CM8—“Community-Engaged Research—Partnership,” to evaluate the collective impact of the RCMI program in engaging communities and contributing to the mitigation of health disparities through research and education.

## 4. Results

### 4.1. Geographic Distribution and Target Populations

**Figure 1:**
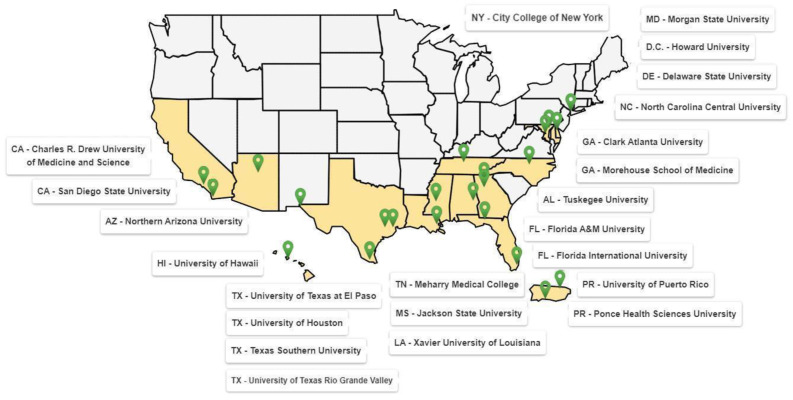
Geographical location of the RCMI U54s. States highlighted in yellow indicate those with active U54 RCMI program sites, while green droplet icons denote the specific geographic locations of each site. Many centers operate in states with historically limited investment in health equity initiatives, serving populations experiencing significant health disparities.

In 2024, RCMIs operated in 46 communities across 11 states, the District of Columbia, and Puerto Rico (Figure 1). Based on data from the Signature Program Monograph, the RCMIs targeted communities with disproportionate health inequities, described by one or more of the following subpopulations: 14 targeted Hispanics, 13 African Americans, 2 Asian Americans, 4 Indigenous Americans, 7 rural, and 6 urban [12]. Combining this data with data derived from the NIH Reporter, the target populations and communities served and engaged by the RCMI U54 Centers through the CECs can be broadly categorized into the following five distinct classifications [13]:

1. Ethnically diverse, less resourced minority communities in urban areas in places like the DC metropolitan area, East Baltimore, and the Greater Houston Community.
2. Hispanic/Latinx and Caribbean communities of various heritages in South Florida, Southern and Western Puerto Rico, and El Paso, Texas.
3. Low-resourced African Americans or those experiencing poor health in the Black Belt and rural areas of Alabama, Nashville, Tennessee, and Atlanta, Georgia.
4. Native Hawaiian and other Pacific Islander (NHOPI) communities in Hawaii.
5. Populations experiencing inadequate healthcare, such as persons of color, in parts of the country, such as Kent and Sussex counties in Delaware, and Central Mississippi.

### 4.2. Descriptors of Key Functions of the CECs Expressed as Keywords

**Figure 2:**
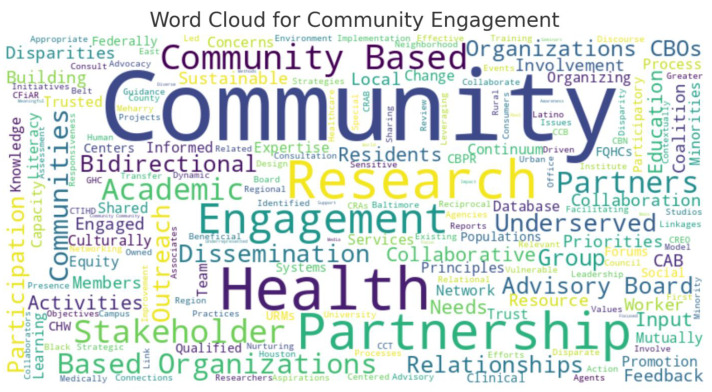
Word Cloud for Keywords Related to Community Engagement. Word size and boldness reflect frequency; ‘Community’ is the most prominent term.

**Figure 3:**
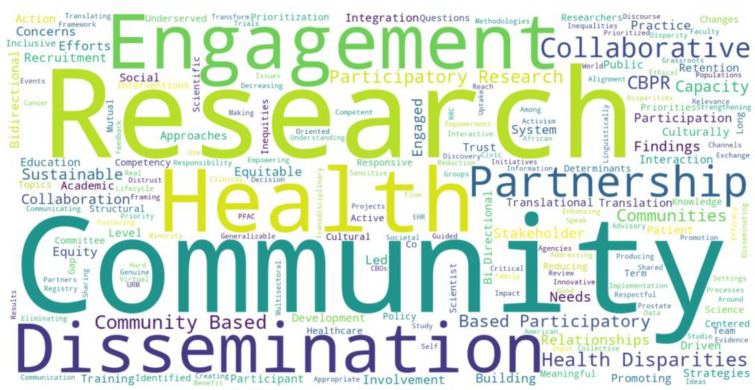
Word Cloud for Keywords Related to Community-Based Participatory Research (CBPR) Principles.

Figure 2 and Figure 3 illustrate keywords derived from RCMI CEC reports, highlighting central areas of focus: community engagement, research, health, partnerships, and outreach. While these visuals reflect the scope of activity, they do not fully capture the nature or depth of engagement.

To provide a more accurate account of engagement intensity, the CEC activities can be contextualized using the CDC’s Spectrum of Community Engagement, from outreach and consultation to collaboration and shared leadership. While several CECs engage in bidirectional partnerships consistent with CBPR principles, many activities fall within the consultation or involvement levels of the spectrum. For example, the University of Houston CAB collaborates with the Community Research Advisory Board to assess community health priorities, guide research emphases and design, and ensure a trusted presence in the community. Morehouse School of Medicine CEC supports the establishment of Patient and Family Advisory Committees (PFACs) for specific projects to address implementation challenges during the project’s lifecycle. The University of Texas at El Paso collaborated with local agencies to host a large health fair that included blood pressure screenings and partnered with the El Paso Diabetes Center to offer HbA1C testing and disseminate information about type 2 diabetes. Table 1 provides a tabular summary of the CDC’s Spectrum of Community Engagement model.

Moving beyond keyword summaries, it is essential to highlight outcomes associated with engagement efforts, such as new and sustained community participation, shifts in institutional practice, or the development of co-created interventions.

### 4.3. Community Engagement Strategies

Across the 21 RCMI CECs, 135 unique strategies were identified and synthesized into 15 thematic areas and five major categories. These strategies reflect a range of approaches to advancing health equity by fostering trust, collaboration, and partnerships between researchers and communities with significant health needs. The depth of engagement varies, and while some initiatives reflect CBPR principles, many align with mid-level engagement (consultation and involvement) as outlined in the CDC framework. Importantly, priority areas were not solely determined by investigators. Across centers, community input was integrated through needs assessments, routine CAB feedback, and ongoing evaluation processes, ensuring that engagement strategies reflected issues of shared importance. Table 2 provides a tabular summary of the major and sub-themes operationalized into unique activities.

Where possible, sites reported early indicators of success, such as increased enrollment in health studies, enhanced researcher trust ratings among community participants, or policy changes influenced by community feedback.

### 4.4. Dissemination Strategies

To ensure that research findings reach and resonate with their intended audiences, CECs employed a range of dissemination strategies, categorized in Table 3. The summary also provides high-level descriptors of each RCMI’s specific dissemination activities for the target population(s) served.

### 4.5. Lessons Learned from Community-Engaged Signature Programs

Utilizing data from the RCMI CEC Signature Programs, nine themes emerged from their lessons learned [12]. The themes highlight the importance of trust and transparency in building partnerships, the need for flexible infrastructure to respond to community input, the value of culturally tailored approaches and recognizing community expertise, and the utility of evaluation in refining strategies over time. Table 4 summarizes the nine themes and specific examples articulated by the CECs. Overall, sites emphasized that sustainable community engagement depends on mutual respect, shared benefit, and institutional responsiveness to community priorities.

RCMIs have learned that success depends on strategic partnerships, capacity building, and mutual respect for researchers’ and communities’ expertise and needs.

### 4.6. Preliminary Findings of Evaluation of Community Engagement Metrics

From 2022 to 2023, the RCMI CEC network engaged 405 community partners, with a median of 25 partners per center. Among these, 79 (19.5%) were formalized through Memoranda of Understanding (MOUs) or subawards. Centers reported forming 44 new partnerships during this period. The longevity of these partnerships varies, with 382 partners engaged for 2–5 years, 25 for 6–10 years, and 16 for more than a decade. Additionally, 38 partnerships are directly linked to funded projects, with a median of one per center. During the same period, 57 active community-engaged research projects were recorded, averaging three per site. Among these, 19 projects were led by minority principal investigators (PIs), and 15 by early-stage investigator (ESI) PIs. 31 projects are linked to RCMI Pilot Projects funding, demonstrating the consortium’s role in supporting early-stage research initiatives. Four received external funding.

## 5. Discussion

The RCMI CEC consortium was developed to enhance community-engaged health disparities research by standardizing and strengthening best practices across institutions. Key achievements include the widespread adoption of Community Engagement (CE) best practices, accelerated onboarding of newly funded RCMIs, and enhanced academic–community partnerships and relationships. The Consortium has leveraged community assets to expand outreach, boost study participation, and promote national dissemination of RCMI activities. By improving access to CE resources and enhancing the community engagement skills of investigators, the Consortium has elevated the overall quality and impact of health disparities research (HDR). This coordinated approach has also increased the visibility of RCMI investigators and improved efficiency in translating research into community benefit, advancing the shared mission of addressing structural health inequities of the RCMI.

### 5.1. Target Populations

RCMI U54 programs serve diverse populations across urban, rural, and island communities. These populations are categorized into six groups: Ethnically diverse, minority communities in urban areas; Hispanic/Latinx and Caribbean; African American communities experiencing health disparities; Native Hawaiian and other Pacific Islander (NHOPI) communities; communities with limited access to medical care; and rural populations, highlighting the program’s significant role in addressing health disparities. RCMI research priorities reflect population needs: 10 sites engaged in cancer research; 6 in nutrition and food security; 13 in clinical trials; 9 in COVID-related studies; and 5 in non-disease-specific research [12]. For Clinical and Translational Science Awards (CTSAs), the initiatives related to community engagement are primarily centered on establishing relationships with community representatives associated with various organizations that have access to populations underrepresented in clinical trials [16,17]. Disaggregated research in NHOPI communities provides culturally relevant data previously obscured by aggregation with Asian American groups [18,19]. Research in rural communities, particularly among those with limited access to medical care, deepens the understanding of how non-medical determinants of health—such as access to food, housing, and transportation—influence health [20]. These efforts challenge assumptions about the capacity of communities affected by health disparities to participate meaningfully in research, reframing them as essential contributors to advancing health equity [21]. 

### 5.2. Summary of the CEC Functions

This analysis addresses gaps in the literature by documenting, at a national scale, how CECs operationalize engagement across diverse communities, and by synthesizing practices that have not previously been compared across institutions. Word clouds generated from CEC documentation highlight terms like “community,” “health,” “partnership,” and “research.” These descriptors align with CEC priorities, but they offer limited insight into the depth of engagement or outcomes. Using the CDC Spectrum of Community Engagement as a framework, CEC activities range from outreach to shared leadership. While CBPR principles guide many strategies, engagement intensity varies by site and initiative. The prevalence of terms such as “engagement,” “dissemination,” and “partnership” reinforces the central role of community in RCMI’s mission. However, quantitative data and concrete examples of outcomes are needed to contextualize impact.

Unlike informal partners, such as community members engaged through forums or outreach events, who provided critical feedback and contextual insights without defined scopes of work, formal partners—such as CAB members, community-based organizations, or faith-based groups with contracts or subawards—had ongoing responsibilities including reviewing study materials, shaping research priorities, and participating in governance. While the prevalence of informal partnerships allowed flexible participation and broad community input, this structure may limit opportunities to secure external funding or sustain long-term projects. Indeed, only 4 of the 57 community-engaged research projects received external funding. These patterns underscore the need for additional structural support, formalized partnerships, and investment to enhance the sustainability, scope, and impact of community-engaged research.

### 5.3. Community Engagement Strategies

Engagement with the community must extend beyond recruitment to include collaboration in planning, implementation, and evaluation. Tindana et al. and Israel et al. emphasize the value of incorporating community input throughout the research process [22,23]. 

Building on the 135 strategies identified across the 21 RCMI CEC sites, the following discussion highlights five overarching themes that capture the shared approaches and lessons learned. The “Access and Equity” strategy emphasizes the importance of inclusivity and representation in research endeavors; this aligns with one of the principal goals of the CTSAs engaging communities [16,17,24]. “Capacity Building and Empowerment” strategy promotes academic–community collaboration that fosters mutual learning and shared leadership between researchers and communities [6]. The “Outreach and Communication” strategy enhances the recruitment and retention of diverse populations in clinical research and helps design culturally tailored health interventions that resonate with targeted communities [25]. The “Partnerships and Collaboration” strategy addresses shared decision-making and leadership, as well as the enhancement of community involvement and trust, ensuring that research agendas align with community priorities and promote the significant uptake of outcomes [26]. Under the “Research and Knowledge Sharing” strategy, CBPR fosters equitable collaboration in study design and dissemination, while culturally relevant communication enhances the impact of research [27,28]. These strategies reflect a continuum of engagement approaches, many of which are aligned with CBPR principles and the CDC’s Spectrum of Community Engagement, summarized in Table 1. Though progress is being made, the aspect of shared leadership or governance in the academic–community partnership still needs improvement. Additionally, resource sharing is another area that requires strengthening. In practice, resource sharing also encompasses funding equity, with CECs experimenting with contracts, stipends, and budget allocations to ensure community organizations have more equitable access to research funds. While academic investigators often receive larger allocations tied to institutional appointments, several CECs have piloted dedicated budget lines and subcontracting mechanisms to promote greater funding equity between partners. Persistent institutional barriers remain, but these efforts represent important steps toward parity.

### 5.4. Dissemination Strategies

Effective dissemination is essential for ensuring that research findings reach and benefit target populations. In their article titled “Ten Simple Rules for Innovative Dissemination of Research,” Ross-Hellauer et al. present several rules that align with the six key themes of dissemination strategies used by the RCMI CECs. Specifically, rules 5 (Remix Traditional Outputs), 7 (Think Visual), and 8 (Respect Diversity) correspond with these themes: Accessible and Community-Centered Communication, Direct Engagement and Dialogue, Leveraging Digital and Mass Media for Broader Outreach, Culturally Tailored Communication, Mixed Media and Hybrid Approaches, and Incentivizing Dissemination Efforts [29]. The dissemination strategies employed by the RCMI CECs align with Rules 5, 7, and 8 of Ross-Hellauer et al.’s framework. Rule 5, Remix Traditional Outputs: RCMIs enhance research dissemination through summaries, media engagement, and digital platforms, expanding reach and impact. Leveraging conferences, repositories, and social media further boosts visibility and engagement, aligning with the RCMI CEC strategies under Accessible and Community-Centered Communication and Leveraging Digital and Mass Media for Broader Outreach.

Rule 7, Think Visual: RCMIs use visuals such as data visualizations, infographics, comics, and video abstracts to make complex concepts more accessible and engaging. Creative methods, including science, art, theater, and dance, also help expand reach and impact. This aligns with several RCMI CEC strategies under the domains of Accessible and Community-Centered Communication, Direct Engagement and Dialogue, Culturally Tailored Communication, and Mixed Media and Hybrid Approaches.

Finally, Rule 8, Respect diversity, addresses the importance of diversity in academic discourse, covering aspects such as gender, ethnicity, and digital literacy. It highlights the importance of inclusiveness in research. The 2017 Progression Framework urged the development of a diversity strategy, the promotion of inclusive messaging, and the incorporation of diversity into scientific activities, education, and training [30]. This aligns with the domains of Incentivizing Dissemination Efforts and Leveraging Digital and Mass Media for Broader Outreach.

### 5.5. Lessons Learned from Community-Engaged Signature Programs

Nine cross-cutting themes emerged from the 21 Signature Programs. Trust: As supported by studies such as that of Lansing et al., the concept of building trust in research is essential for fostering healthy, transparent, and reciprocal relationships, creating safe environments, and promoting community capacity building [8]. Equity: Most of the signature programs developed and implemented by the CECs focus on equity, aligning with recommendations to ensure fair participation and benefits while minimizing exclusivity, which can exacerbate existing disparities [9]. Cultural Sensitivity: Two of the lessons learned pertaining to “Tailoring Approaches to Cultural and Community Needs” aligned with five of the six forms of cultural capital articulated under Yosso’s Community Cultural Wealth (CCW) Applying framework (2005), while including cultural sensitivity, which is critical in promoting health equity and authentic relationships with diverse community partners [10,11]. Strong Infrastructure: Effective engagement of communities in research requires a robust infrastructure that provides the necessary resources and systems to promote equitable infrastructure choices, ensuring that community needs are met [31]. This finding aligns with the CEC signature programs’ mission to create a strong infrastructure for community engagement. These lessons affirm that building durable academic–community relationships depends on intentional design, responsiveness to community priorities, and shared investment in outcomes.

### 5.6. Strengths and Limitations

Strengths: This study presents a consolidated analysis of strategies from 21 CECs, offering insight into shared practices for community engagement in health disparities research. It highlights common data elements to evaluate engagement, health promotion, education, and impact--extending beyond individual site accomplishments to reveal collective trends across the RCMI network

Limitations: Most data were obtained from NIH RePORTER, which may lack contextual nuance or real-time updates. However, with most sites funded for more than two five-year cycles, there is high confidence (90% CI) in the stability and implementation of their core activities. The key limitation is the absence of direct data from community partners or community advisory boards, which would have provided more robust insight into community perceptions and outcomes.

## 6. Conclusions

The Research Centers in Minority Institutions (RCMI) program is a strategic NIH initiative aimed at building research capacity at institutions that primarily serve underrepresented populations. Through investments in infrastructure, biomedical workforce development, and community partnerships, RCMIs play a crucial role in mitigating health disparities by addressing the medical and non-medical needs of populations through the biomedical research enterprise. The program deepens our understanding of how to conduct health disparities research that improves access, outcomes, and trust in science. While many RCMI sites incorporate principles of Community-Based Participatory Research (CBPR), the level of engagement varies. What remains consistent is a commitment to grounding research in community priorities, enhancing academic–community collaboration, and building systems that sustain meaningful engagement. Given the changing federal priorities for the Research Centers in Minority Institutions (RCMI) Program, it is crucial for funded Centers to reassess their site-level activities and goals. This review will help ensure they align with current national priorities. The RCMI program’s Community Engagement Cores (CECs) play a key role in supporting NIH goals by boosting research capacity at minority-serving institutions to combat health disparities. By building sustainable community partnerships and developing the biomedical workforce, RCMI centers align their strategies with national health equity objectives, including those of “Make America Healthy Again.”

Key strategies include promoting access to and equity in research, building academic–community research capacity, enhancing outreach and communication, fostering collaborative partnerships, and effectively disseminating valuable knowledge. The success of the RCMI program relies heavily on the strength of its Community Engagement Cores, which prioritize trust-building, cultural relevance, and sustainable infrastructure for engagement. These efforts lay the foundation for long-term, community-driven change and contribute to improved health outcomes among some of the nation’s most vulnerable populations.

## Figures and Tables

**Table 1:** Components of the CDC’s Spectrum of Community Engagement.

Level	Community Role
Outreach	Informed: One-way communication. Information is provided to the community.
Consultation	Asked: Two-way communication. Community feedback is requested and considered.
Involvement	Involved: Community participates in planning and implementation. Their input influences decisions.
Collaboration	Partnered: Shared decision-making. Community members and organizations are equal partners.
Shared Leadership	Shared Decision-Making: Joint leadership between the community and institutions.
Community Ownership	Leads and Owns Decisions: The Community leads the effort. Organizations support but do not direct.

**Table 2:** Tabular Summary of Distinct Community Engagement Strategies Employed by the CECs.

Major Themes	Themes	Themes Operationalized as Activities
Access and Equity	Reducing Barriers to Clinical Research Participation:	Address trust issues and barriers to participation through education forums and transparent communication.
		Promote participation in clinical trials and other research studies.
		Example: The University of Texas at El Paso utilizes Community Health Workers to recruit participants for clinical research because the CHWs are trusted and speak to participants at a level they can understand.
	Evaluation and Monitoring of Community Engagement Activities:	Develop logic models and evaluation tools to measure the success and impact of community engagement activities.
		Use formative and summative evaluations to guide improvements.
		Example: Northern Arizona University’s evaluation of its Community-Campus Partnership Support demonstrated that 75% of funded teams agreed that their project helped build capacity to engage in health equity research together.
Capacity Building and Empowerment	Needs Assessments and Feedback Mechanisms:	Conduct community-level assessments to identify health needs, barriers, and resources.
		Use surveys and feedback from community partners to shape health-related priorities.
		Example: Texas Southern University uses surveys and community input to identify health-related concerns in the Greater Houston Community.
	Developing Databases and Hubs for Community Resources:	Create centralized databases of community resources, services, and outreach activities.
		Develop information hubs to streamline access to community engagement efforts.
		Example: The Rural Health Hub at North Carolina Central University fills a critical need for access to health information, nutritious food options, and other wellness services for people living in Halifax County.
	Training and Capacity Building:	Provide training for researchers on community engagement and health equity.
		Build capacity among community members to participate in research.
		Example: Through its Student Research Training Program, Morehouse School of Medicine paired its researchers with undergraduate students from four local institutions for up to two years.
Outreach and Communication	Health Education and Outreach Programs:	Offer culturally sensitive health education seminars and workshops based on identified community needs.
		Develop programs to promote cancer screening, health literacy, and nutrition education.
		Example: Xavier University of Louisiana developed culturally and linguistically tailored educational programs, such as the Cancer 101 Education Program and the RCMI Cancer Health Advisor Program, that empower community members through monthly training on cancer.
	Community Forums and Events:	Host community forums, topical workshops, and town halls to address pressing health issues collaboratively.
		Facilitate “town and gown” events to bridge the gap between research and community stakeholders.
		Example: At the height of COVID-19, Florida International University hosted 7 town halls to disseminate vaccine literacy materials to over 1800 people from more than 78 zip codes throughout South Florida.
	Engagement Through Social and Mass Media	Utilize social media platforms and mass media outlets to raise awareness about research and community programs.
		Example: Healthy Disruptions, a podcast launched by the University of California, Riverside, highlights how community members and researchers are collaborating towards action-based solutions.
Partnerships and Collaboration	Developing Community Partnerships:	Establish partnerships with community-based organizations (CBOs), healthcare systems, Federally Qualified Health Centers (FQHCs), and other local agencies.
		Leverage longstanding partnerships for outreach and engagement.
		Example: Texas Southern University leverages established partnerships with over 18 Faith-Based Organizations (FBOs) and 24 Community-Based Organizations (CBOs) in health education, conference planning, and information sharing.
	Establishing Community Advisory Committees:	Form community consultation councils or advisory boards to provide input and feedback.
		Create patient and family advisory committees (PFACs) to assist with project implementation.
		Example: The relationships established through Florida International University’s CAB network provide insight into best practices for community engagement, and participant recruiting/retention, guide the dissemination of research results, and facilitate new joint community-academic grant submissions
	Promotores de Salud/Community Health Worker (CHW) Models:	Engage CHWs to conduct grassroots outreach and connect with populations experiencing health disparities.
		Use CHWs to deliver health education and facilitate research participation.
		Example: The community health workers at the University of Texas at El Paso’s Border Biomedical Research Center conducted outreach to 210,761 individuals who received COVID-19 information.
	Binational and Bidirectional Partnerships:	Establish partnerships across borders to address shared health concerns, particularly in regions with high concentrations of immigrants.
		Example: University of Puerto Rico Medical Sciences Campus hosts seminars and workshops aimed at fostering dialogue and knowledge exchange in community-based participatory research, citizen science, and strategies for effectively communicating research results to broader audiences.
Research and Knowledge Sharing	Community-Based Participatory Research (CBPR)	Use CBPR methods to co-develop and conduct research with community partners.
		Include community members in the research review process.
		Example: The Morgan CARES award at Morgan State University is reviewed by a diverse steering committee that includes representatives from both community and academia.
	Dissemination of Research Findings:	Disseminate research findings in culturally relevant and accessible formats, such as lay-language reports or multimedia.
		Partner with community stakeholders to develop effective dissemination strategies.
		Example: The University of Hawaii at Manoa ensures that 100% of pilot applicants provided lay summaries along with their abstracts and full proposals, which facilitated review by the community.
	Facilitating Researcher-Community Interactions:	Foster opportunities for researchers to engage with community members through workshops and collaborative activities.
		Example: Jackson State University leveraged organizations like the 100 Black Men of Jackson, Mississippi Community Health Workers, and the Mississippi State Department of Health to provide resources and access to priority populations.

**Table 3:** Tabular Summary of Distinct Dissemination Strategies Employed by the CECs.

Domains	Distinct Dissemination Strategies
Accessible and Community-Centered Communication	Community-Friendly Materials: CECs use accessible formats, such as social media, newsletters, health brochures, and videos, to disseminate research findings
	Reports and Publications: Many CECs utilize reports, newsletters, and social media to disseminate their findings to both community and academic audiences.
Direct Engagement and Dialogue	Town Hall Meetings/Community Forums: These platforms are used for open discussions and the dissemination of research findings, ensuring direct community engagement.
	Multi-Media and Face-to-Face Contact: Some CECs, such as UTEP BBRC-HCHD, combine multimedia technology and in-person communication to reach underserved communities and foster trust.
Leveraging Digital and Mass Media for Broader Outreach	Media and Technology: Digital platforms, including social media, webinars, and online forums, are utilized to reach a broader audience.
	Social Media and Technology: Digital platforms are extensively used for spreading research findings and engaging wider audiences.
	Social and Mass Media: TSU utilizes social and mass media (e.g., newspapers, Radio, etc.) to spread awareness and organize events that bridge the gap between research findings and the community.
Culturally Tailored Communication	Culturally Relevant Methods: FAMU employs culturally relevant methods, including community health worker models, educational materials, and direct communication, to ensure that research is accessible and tailored to local communities.
Mixed Media and Hybrid Approaches	Multi-Media and Face-to-Face Contact: Some CECs, such as UTEP BBRC-HCHD, combine multimedia technology and in-person communication to reach underserved communities and foster trust.
Incentivizing Dissemination Efforts	Dissemination Awards and Incentives: DSU incorporates programs, such as Dissemination Awards, to incentivize impactful communication and ensure that dissemination activities are contextually relevant.

**Table 4:** Themes of Lessons Learned in the Implementation of CEC Signature Programs.

Themes	Examples of Uniquely Cited Lessons Learned
Co-Participation	
Building and Sustaining Trust and Partnerships	Trust is foundational for effective community engagement and research partnerships.
	Partnerships with trusted organizations, including faith-based groups and community leaders, increase engagement and credibility.
	Long-term, mutually beneficial relationships require consistent engagement, transparency, and recognition of community contributions.
Capacity Building for Communities and Researchers	Training community-based organizations (CBOs) in research practices and strengthening their capacity fosters sustainable collaborations.
	Workshops and mentorship programs help researchers and communities navigate challenges and align on research goals.
	Involving students and early-career researchers in community-engaged activities helps grow the next generation of community-focused professionals.
Bidirectional and Equitable Engagement	Communities must be engaged as equal partners, with their expertise and contributions acknowledged and respected.
	Bidirectional relationships enable researchers and communities to share knowledge and co-develop solutions.
	Allowing community leaders to guide discussions and priorities fosters more meaningful and impactful engagement.
Prioritizing Community	
Tailoring Approaches to Cultural and Community Needs	Effective strategies must incorporate culturally and linguistically appropriate methods to address the unique needs of diverse populations.
	Understanding and respecting cultural practices, values, and histories enhances trust and engagement.
Leveraging Local Gatekeepers and Community Experts	Community health workers (CHWs), community coalitions, and advisory boards are essential for bridging gaps between researchers and underserved populations.
	Involving community stakeholders early in the research process helps align objectives and improves recruitment.
Empowering and Recognizing Community Contributions	Recognizing and celebrating community contributions, even in non-monetary ways, fosters goodwill and continued collaboration.
	Creating community platforms to share priorities and experiences strengthens engagement and mutual understanding.
Addressing Operational Infrastructure	
Importance of Strategic and Flexible Infrastructure	Dedicated infrastructure, including committees, guidelines, and trained personnel, is critical for supporting engagement efforts.
	Flexibility and responsiveness to the community and researchers’ needs improve collaboration outcomes.
Addressing Institutional and Bureaucratic Barriers	University bureaucratic processes often hinder timely community engagement and resource distribution, necessitating innovative solutions such as partnering with nonprofits to manage funds.
	Streamlining administrative processes and providing extensive support for community engagement improve efficiency and responsiveness.
Evaluation and Continuous Improvement	Integrated evaluation processes ensure that community engagement activities are effective and sustainable.
	Feedback from community members and advisory boards drives improvements and fosters innovation in programs.

## Data Availability

Ninety percent of the data analyzed for this paper were ascertained from the NIH RePORTer.
